# Noninterventional Open-Label Trial Investigating the Efficacy and Safety of Ectoine Containing Nasal Spray in Comparison with Beclomethasone Nasal Spray in Patients with Allergic Rhinitis

**DOI:** 10.1155/2014/297203

**Published:** 2014-05-28

**Authors:** Uwe Sonnemann, Marcus Möller, Andreas Bilstein

**Affiliations:** ^1^Private Health Centre, Institute for ENT Elmshorn, Hermann-Ehlers-Weg 4, 25337 Elmshorn, Germany; ^2^Joint Practice for ENT, Willy-Brandt Straße 2, 21335 Lüneburg, Germany; ^3^Bitop AG, Stockumer Straße 28, 58453 Witten, Germany

## Abstract

*Objectives*. The current study aimed to compare the efficacy and safety of a classical anti-inflammatory beclomethasone nasal spray in comparison to a physic-chemical stabilizing ectoine containing nasal spray in the treatment of allergic rhinitis. *Design and Methods*. This was a noninterventional, open-label, observational trial investigating the effects of beclomethasone or ectoine nasal spray on nasal symptoms and quality of life. Over a period of 14 days, patients were asked to daily document their symptoms. Efficacy and tolerability were assessed by both physicians and patients. *Results*. Both treatments resulted in a significant decrease of TNSS values. An equivalence test could not confirm the noninferiority of ectoine treatment in comparison with beclomethasone treatment. Although clear symptom reduction was achieved with the ectoine products, the efficacy judgment showed possible advantages for the beclomethasone group. Importantly, tolerability results were comparably good in both groups, and a very low number of adverse events supported this observation. Both treatments resulted in a clear improvement in the quality of life as assessed by a questionnaire answered at the beginning and at the end of the trial. *Conclusion*. Taken together, it was shown that allergic rhinitis can be safely and successfully treated with beclomethasone and also efficacy and safety were shown for ectoine nasal spray.

## 1. Introduction

Allergic rhinitis is a common disease with estimated 600 million patients suffering from this disease worldwide [1]. According to a large scale investigation, about 20% of the European population suffers from allergic rhinitis [2] and numbers are increasing, particularly in industrial states. Although not being a life-threatening disease, allergic rhinitis has a considerable impact on general well-being and work/school performance, and particularly its impact on comorbidities such as, for example, asthma reflects the need for good treatment options.

A number of pharmacological treatments against allergic rhinitis exist, such as antihistamines, leukotriene receptor agonists, mast cell stabilizing agents, and glucocorticosteroids. According to the ARIA (Allergic Rhinitis and its Impact on Asthma) guidelines, intranasal glucocorticosteroids are recommended as pharmacological treatment of allergic rhinitis and should be prescribed preferable to intranasal antihistamines and oral leukotriene receptor agonists [1]. However, many patients have reservations to use corticosteroids, and phobia of their usage can result in bad compliance [3]. This together with the fact that patients often seek alternative treatments to pharmacological options reflects the need for new treatment strategies.

The current noninterventional trial compared efficacy and safety of treatment of allergic rhinitis patients with intranasal spray containing either the glucocorticoid beclomethasone or the natural, nonpharmacological substance ectoine. Overview of the results are shown in Figure 9.

Ectoine is a compatible solute which is produced by microorganisms living under harsh environmental conditions such as extreme salinity or dryness [4–6]. In those microorganisms, ectoine acts as natural cell protectant. Halophilic microorganisms living in habitats of high ionic strength cope with hyperosmotic stress by changing the composition of membrane lipids and by regulating the intracellular concentration of low molecular weight solutes such as the compatible solute Ectoine. As a result of the latter response, the cells are able to maintain proper osmotic balance under conditions of hyperosmotic stress, which is crucial to prevent the cell from leaking water, hence avoiding irreversible plasmolysis and dehydration, and to generate turgor pressure within limits necessary for growth [7, 8]. Ectoin is industrially produced via the “bacterial milking process” using the gram negative bacterium* Halomonas elongata* [9, 10]. It is known that ectoine works via an entropy-driven mechanism called “preferential exclusion” or “preferential hydration” during which ectoine influences the characteristics of the water shell surrounding biomolecules like membranes. By excluding osmolytes from the direct hydrate shell of proteins and membranes, a preferential hydration of such proteins or membranes occurs, thereby stabilizing their native confirmation and making them less vulnerable to external stressors [11, 12]. Preclinical studies have demonstrated that the beneficial effects of ectoine can be transferred to human or animal models, and they have also shown that ectoine possesses membrane-stabilizing and inflammation-reducing properties [13–16]. Additional experiments on human nasal epithelial cell lines have confirmed the protective action of ectoine against osmotic stress (data not published). Recent developments have demonstrated that this attribute can be successfully transferred to a number of application forms such as ectoine containing creams, nasal sprays, or eye drops which can be used on humans for treatment of rhinosinusitis and atopic dermatitis [11, 12] and also congress reports point towards efficacy when applied to patients with allergic rhinitis [17–20].

This study assessed development of nasal symptoms, changes in quality of life, and judgment of efficacy and tolerability upon treatment with either the well-known steroid beclomethasone or barrier stabilizing, physically acting ectoine nasal spray in order to compare the effect levels of these different treatment concepts in patients with allergic rhinitis.

## 2. Materials and Methods

### 2.1. Treatment and Study Design

This was a controlled, open-labelled, noninterventional, multicenter study assessing the efficacy and safety of ectoine containing nasal spray in comparison to beclomethasone containing nasal spray. The patients could freely choose their treatment: they were treated with either ectoine nasal spray or beclomethasone containing nasal spray (0.05% beclomethasone).

According to §23b of the German medical device law, this study was carried out with the CE-marked medical device ectoine nasal spray without changes in its intended use; therefore, §§20–23a of the MPG had not been complied with. Open observational trials are health authority accepted in Germany for nonpharmaceutical treatment options. According to a general statement by ethical committees, this type of study does not allow for a placebo group, because this would involve a lack of benefit for patients.

Ectoine nasal spray is an isotonic solution containing 2% ectoine, natural salt, and water. According to the instructions for use, one puff of the spray had to be applied into each nostril three times daily. The beclomethasone spray was used in accordance with the instructions for use: two puffs of the spray had to be applied into each nostril twice a day. Each puff of the nasal spray contains 0.05 mg beclomethasone diproprionate. Further ingredients are benzalkonium chloride (preservative), polysorbate, glucose, cellulose, sodium carmellose, water, sodium hydroxide, and hydrochloric acid for pH adjustment.

Male and female patients aged 18–70 years with documented diagnosed seasonal allergic rhinitis were eligible for the study, based on the discretion of the investigator. In order to be sure of the allergic symptoms, the nasal symptom score (TNSS) at study start had to be ≥ 6.

Patients had to attend two site visits (V1 on day 0 and V2 on day 14 ± 2). During the entire treatment period, patients were asked to document their symptoms in patient diaries once daily. Assessments of symptoms by physicians were carried out during site visits V1 and V2.

The medication was handed over to the patients by the physician. After completion of the study, no drug accountability was performed.

For simplification reasons, patients of the ectoine group will be termed group 1 in this paper, and patients of the beclomethasone group will be termed group 2.

### 2.2. Endpoints

Primary endpoints were changes in the single symptoms nasal obstruction, rhinorrhea, nasal itching, and sneezing as well as changes in the sum of nasal symptoms (TNSS). Secondary endpoints were the assessment of the symptom itchy ear/palate and assessment of efficacy and tolerability as well as analysis of safety data.

### 2.3. Scoring of Nasal Symptoms

Single nasal symptoms as well as ear/palate itching were assessed with a score described as follows: 0 (no symptoms), 1 (slight symptoms), 2 (moderate symptoms), and 3 (severe symptoms). Patients assessed their symptoms reflectively, and scores describing the symptoms within the last 24 h were documented in the patients' diaries. Physicians scored the current symptoms during both patient visits (V1 and V2).

In order to account for the influence of pollen intensity on the nasal symptoms, the quotient TNSS/pollen count score was calculated. Pollen count scores were derived from online available daily pollen counts for the relevant local area. The scoring of pollen counts was as follows: no pollen count was scored with 0.1, low pollen count with 1, moderate pollen count with 2, and strong pollen count with 3.

### 2.4. Scoring of Efficacy and Tolerability

At the end of the study, patients assessed both efficacy and tolerability with a score of 0 (no efficacy, bad to tolerate), 1 (moderate efficacy, moderate tolerability), 2 (good efficacy, good tolerability), and 3 (very good efficacy, very good tolerability).

### 2.5. Quality of Life Questionnaire

A modified, nonvalidated quality of life questionnaire based on the RQLQ from Juniper et al. was used in this study. During both site visits (V1 and V2), patients were asked to fill out the questionnaire. It contained 14 questions covering three topics (daily life activities, general wellbeing, and emotional status) which were to be answered on a score from 0 (none) to 6 (very/always).

### 2.6. Data Management and Statistics

Data of this open-label trial were collected by the physicians in an anonymized paper CRF and by the patients in diaries and questionnaires. Proper data management was monitored during the study. A SAP was set up before study closure and the data were analyzed according to the SAP. Source data from the CRFs, diaries, and questionnaires were transferred to digital data format by the physicians. The statistical analysis was carried out with SPSS Statistics 20 and SigmaPlot version 12. The primary endpoint TNSS was summarized descriptively for both V1 and V2, and differences between V1 and V2 were documented. Noninferiority of the ectoine product versus the beclomethasone containing spray was assessed with an equivalence range of 15%. Analysis of secondary parameters was done descriptively. In addition, changes during the period of the study were analyzed via Bowker's test of symmetry. Group comparisons were analyzed via Chi-square test or Fisher's exact test. All significance levels were set to 5%. Unavailable data were treated as “missing values.”

## 3. Results

**Figure 1:**
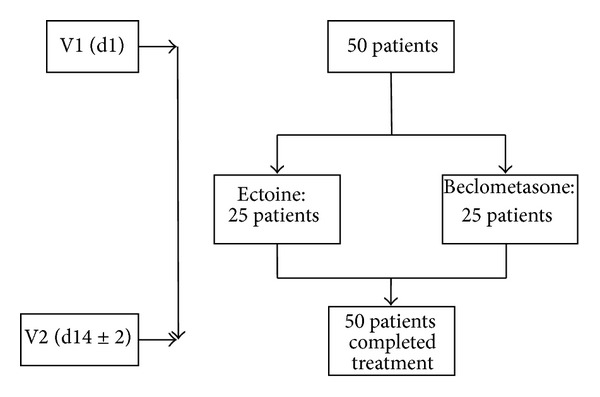
Patient flow during the study. On day 1 (V1), patients were asked to participate in the study. 25 patients received ectoine nasal spray, and 25 patients received beclomethasone nasal spray for a treatment period of 14 ± 2 days. All 50 patients finished the study with the final study visit V2.

The current study was conducted in accordance with the Declaration of Helsinki. All investigations were carried out with the understanding and consent of all participants. The study took part at two German ear nose throat (ENT) practices starting in May 2011 and being completed at the end of June 2011. In total, 50 patients (34 women, 16 men) diagnosed with seasonal allergic rhinitis were included in the study. Mean age of the patients was 33.3 years. Of the 50 patients, 25 received ectoine and 25 patients received beclomethasone nasal spray. All patients completed the study. Distribution of patients is shown in Figure 1.

### 3.1. Total Nasal Symptom Score (TNSS)

The development of the total nasal symptom score (sum of nasal obstruction, rhinorrhea, sneezing, and nasal itching) was judged by both patients and the investigators.

**Figure 2:**
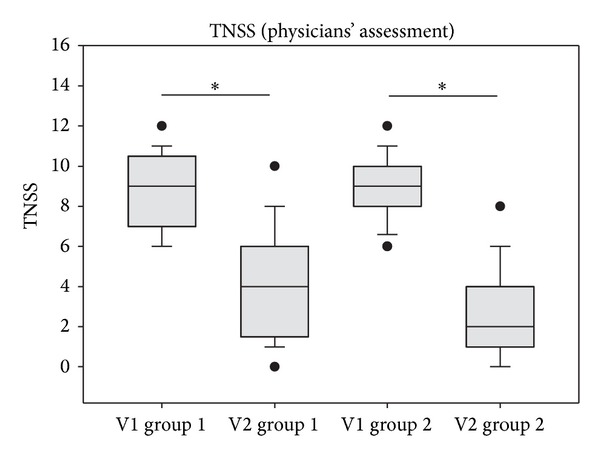
TNSS development during the study based on the physicians' assessment of symptoms. *P < 0.001. Lines within the box mark the median; the upper and lower ends of the box indicate the 75th and 25th percentiles, respectively. Whiskers above and below the box indicate the 90th and 10th percentiles. Dots (•) represent outlying points.

Results of the investigators' assessment are shown in Figure 2. TNSS values decreased significantly in both groups (*P* < 0.001). In the ectoine group, values decreased from 8.76 ± 1.79 (V1) to 4.04 ± 2.75 (V2) corresponding to a total decrease of −4.72 (−51.20%), whereas values in the beclomethasone group decreased from 9.04 ± 1.53 (V1) to 2.52 ± 2.22 (V2) corresponding to a total decrease of −6.52 (−71.49%).

**Figure 3:**
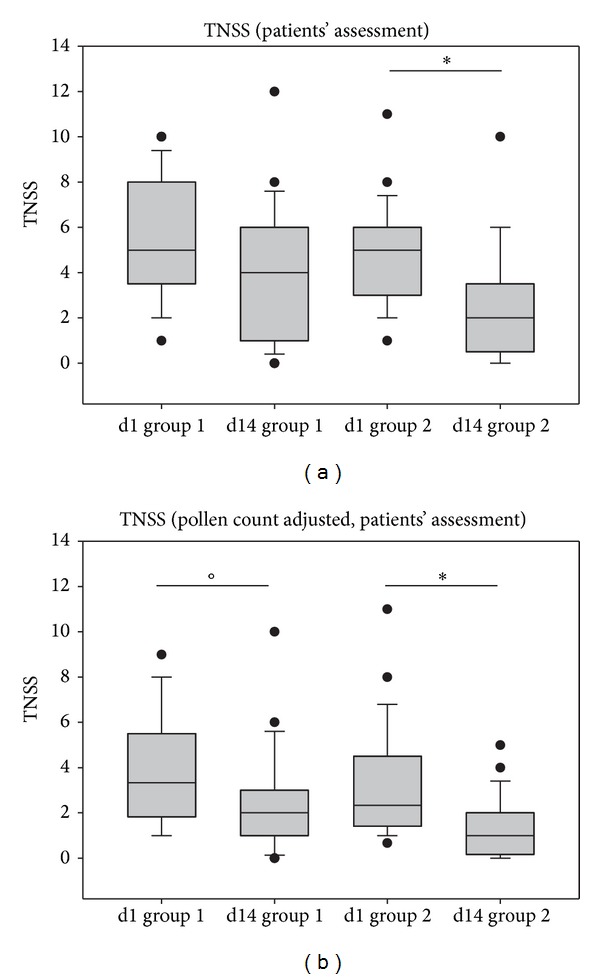
TNSS development during the study based on the patients' assessment of symptoms. (a) TNSS values on day 1 (d1) and day 14 (d14); (b) TNSS values adjusted for pollen counts, *P < 0.001, °*P* = 0.043. Lines within the box mark the median; the upper and lower ends of the box indicate the 75th and 25th percentiles, respectively. Whiskers above and below the box indicate the 90th and 10th percentiles. Dots (•) represent outlying points.

According to the patients' assessment (see Figure 3(a)), TNSS values decreased clearly in the ectoine group (*P* = 0.072, decrease by −12.86%) and a significant decrease was observed in the beclomethasone group (*P* < 0.001, decrease by 39.69%).

In order to consider the influence of pollen on the strength of nasal symptoms, quotients of TNSS values and pollen count scores were calculated. Those confirmed the statistically significant decrease of TNSS values from V1 to V2 as assessed by investigators (*P* < 0.001 for both groups, details not shown). When patients' TNSS values were normalized to the pollen count scores, TNSS decreased in both groups and reached statistical significance (*P* = 0.043 for group 1, *P* < 0.001 for group 2; see Figure 3(b)).

### 3.2. Equivalence Test

An equivalence test was carried out to investigate the hypothesis that ectoine nasal spray is not inferior to beclomethasone containing nasal spray. As shown in Table 1, no significant differences could be shown. Thus, noninferiority of the ectoine nasal spray could not be confirmed.

### 3.3. Comparison of TNSS Values from V1 until the End of the First Treatment Day

**Figure 4:**
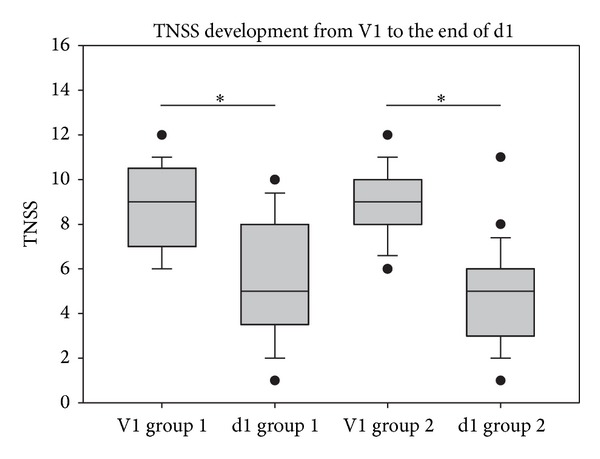
TNSS development from site visit 1 (V1) until the end of the first treatment day (d1). *P < 0.001.

In order to study the time of onset of both treatments, TNSS value development within the first 12 hours of treatment was analyzed. As shown in Figure 4, both groups showed a significant decrease of TNSS values from the first site visit until the first patient assessment at the end of the first day of treatment (*P* < 0.001 for both groups). This indicates that a comparably quick reduction of allergic symptoms was achieved within the first day of treatment in both groups.

### 3.4. Development of Single Symptom Scores

In order to correlate group affiliation and development of single symptoms, data were further analyzed with Fisher's exact test.  Table 2 lists the number of patients with reduced, unchanged, or deteriorated symptoms as assessed by patients themselves or by the physicians. The analysis of data demonstrated that only the patients' assessment of the symptom sneezing revealed a statistically significant correlation (*P* = 0.039), indicating that this symptom improved significantly better in the patient group treated with beclomethasone nasal spray.

### 3.5. Ear/Palate Itching

In addition to the assessment of nasal symptoms, development of ear/palatal itching was assessed both by investigators and by patients. Results are depicted in Table 3 showing that there was no significant correlation between symptom development and group affiliation detectable neither in accordance with the investigators' nor in accordance with the patients' assessment.

### 3.6. Results of the Quality of Life Questionnaire

At the beginning and at the end of study participation, patients were asked to fill out a quality of life questionnaire which consisted of 14 questions. In order to investigate a correlation between changes in life quality and group affiliation, all single parameters of the questionnaire were analyzed via Fisher's exact test. A comparison of the patients' evaluation of quality of life both at d1 and d14 did not show statistical differences between groups 1 and 2 in any of the questions asked (details not shown). Analysis of the results of the quality of life questionnaire as assessed by the investigators only showed a statistical significance (*P* = 0.008) for the parameter “frequency of brushing the nose” indicating that this parameter improved significantly better in the beclomethasone group (for details, see Table 4).

**Figure 5:**
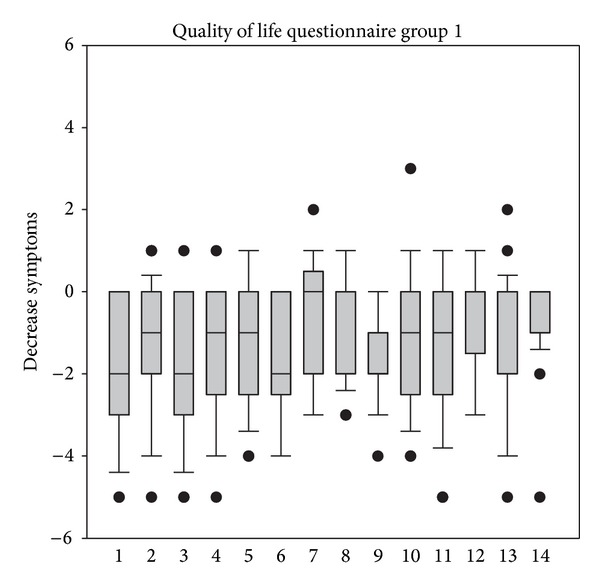
Reduction of scores of the quality of life questionnaire from V1 to V2 in group 1. 1 = frequency of tissue use, 2 = rubbing eyes and nose, 3 = frequency of brushing of nose, 4 = bad sleep, 5 = bad work performance, 6 = fatigue, 7 = thirst, 8 = lack of concentration, 9 = general well-being, 10 = headache, 11 = bad temper, 12 = general disconcertment, 13 = frustration, and 14 = reactions of others to the allergy.

**Figure 6:**
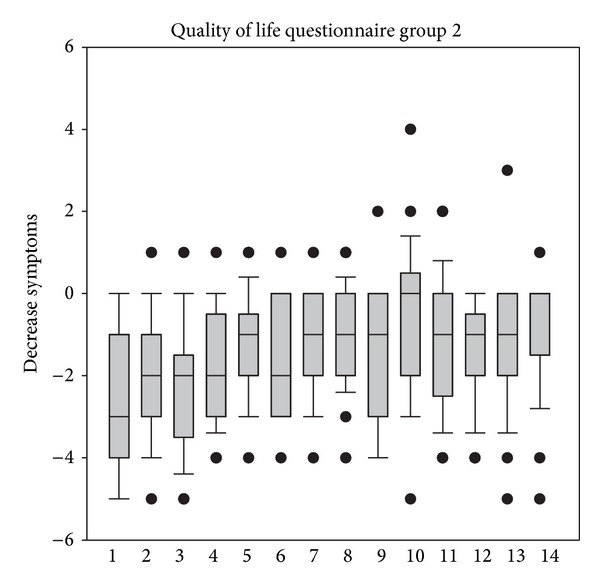
Reduction of scores of the quality of life questionnaire from V1 to V2 in group 1. 1 = frequency of tissue use, 2 = rubbing eyes and nose, 3 = frequency of brushing of nose, 4 = bad sleep, 5 = bad work performance, 6 = fatigue, 7 = thirst, 8 = lack of concentration, 9 = general well-being, 10 = headache, 11 = bad temper, 12 = general disconcertment, 13 = frustration, and 14 = reactions of others to the allergy.

In addition to the analysis described above, total decreases of scores of the quality of life questionnaires were analyzed. As depicted in Figures 5 and 6, treatment resulted in decreases of all questioned parameters, thus indicating that all bothersome points which were covered in the questionnaire of life had improved during treatment.

### 3.7. Efficacy and Tolerability

**Figure 7:**
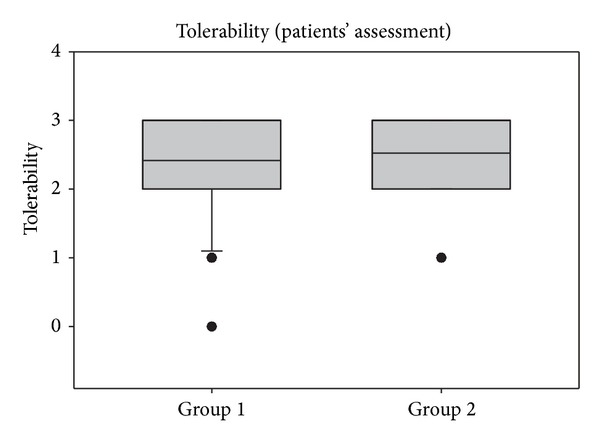
Tolerability assessments of patients during the entire study period of 14 days. Lines within the box mark the median; the upper and lower ends of the box indicate the 75th and 25th percentiles, respectively. Whiskers below the box indicate the 10th percentiles. Dots (•) represent outlying points.

**Figure 8:**
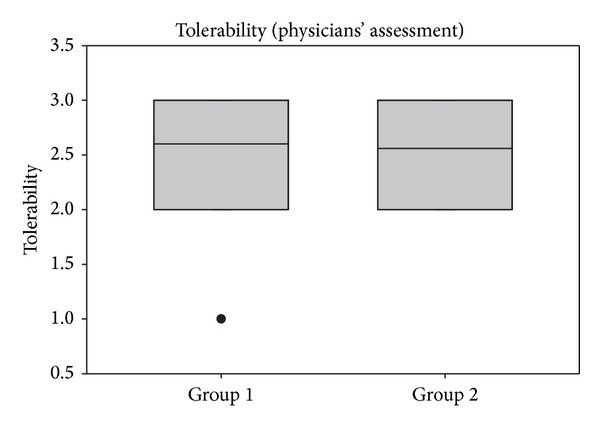
Assessment of tolerability at V2 assessed by the physicians. Lines within the box mark the median; the upper and lower ends of the box indicate the 75th and 25th percentiles, respectively. Dots (•) represent outlying points.

**Figure 9:**
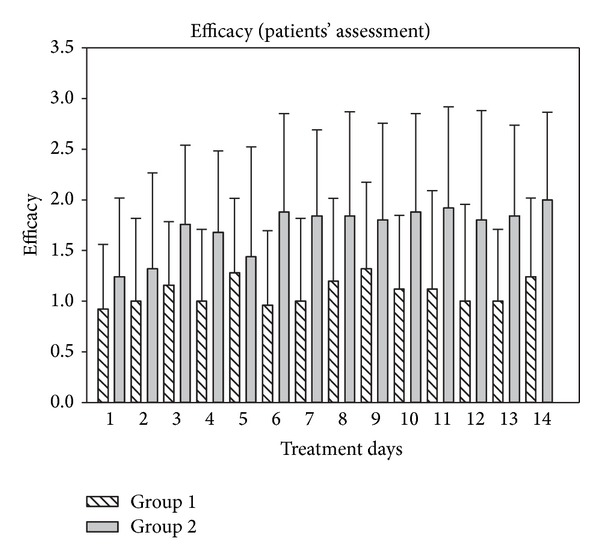
Efficacy assessment of treatments assessed by patients over a study period of 14 days. Mean values ± SD are depicted.

Patients and investigators evaluated both the efficacy and tolerability of treatments during the study. Judgment of patients was given on a daily basis, whereas the investigators assessed those parameters at the end of the study (V2). As shown in Figure 7, patients judged the tolerability of both products similarly, and mean values of 2.42 ± 0.72 (group 1) and 2.53 ± 0.55 (group 2) corresponded to good to very good tolerability. No significant differences were detectable between groups. Similarly, assessment by the investigators during V2 confirmed a good tolerability of the treatments which was comparable between groups (see Figure 8).

**Figure 10:**
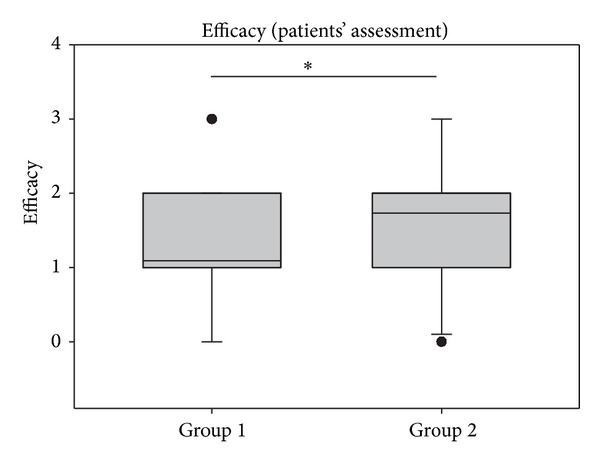
Assessment of efficacy of treatments in groups 1 and 2 over a period of 14 days (mean). *P < 0.001. Lines within the box mark the median; the upper and lower ends of the box indicate the 75th and 25th percentiles, respectively. Whiskers above and below the box indicate the 90th and 10th percentiles. Dots (•) represent outlying points.

**Figure 11:**
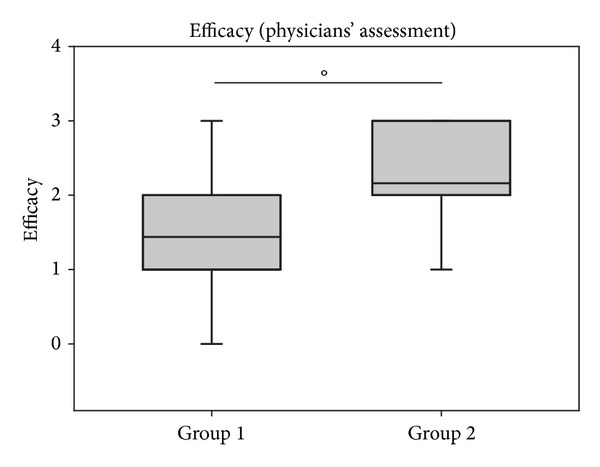
Assessment of efficacy of both treatments at site visit 2 (V2) by investigators. °*P* = 0.009. Lines within the box mark the median; the upper and lower ends of the box indicate the 75th and 25th percentiles, respectively. Whiskers above and below the box indicate the 90th and 10th percentiles. Dots (•) represent outlying points.

The results of the assessment of efficacy of both treatments are depicted in Figures 10 and 11. As shown in Figure 10, efficacy assessment was similar during the first two days of treatment but increased over the treatment period of 14 days in the beclomethasone group compared to the ectoine group. In group 1, mean values of 1.09 ± 0.78 (mean values of entire study period) reflected moderate efficacy assessed by patients and a value of 1.44 ± 1.00 showed similar judgment by the physicians. In group 2, the efficacy was judged as good by patients (1.73 ± 0.94) and as very good by investigators (2.60 ± 0.58).

### 3.8. Adverse Events (AEs)

In total, 3 adverse events were reported. Details are given in Table 5. No serious adverse events (SAEs) occurred during the study. Both AEs occurring in the ectoine group were assessed as highly unlikely by the investigators, whereas the correlation of the AE “burning of nose” with the study medication was judged as probable in the beclomethasone group.

## 4. Conclusions

The current noninterventional, open-label study investigated treatment of allergic rhinitis comparing the intranasal glucocorticoid beclomethasone with that of ectoine containing nasal spray. Within the study, different mode of action, on the one hand the glucocorticoid, was compared to a physical, membrane stabilizing molecule. Importantly, it was shown that nasal symptom scores of both treatment groups improved significantly over the study period of 14 days. Although advantages of the beclomethasone spray in comparison with the ectoine spray were shown, results of the ectoine group showed its potential clinical efficacy. Glucocorticoids bind to specific glucocorticoid receptors which are present on almost all cells of the body. Following binding, transcription of a number of inflammatory cytokines and chemokines can be modulated, which in turn results in decreased recruitment and activation of inflammatory cells [21]. In allergic rhinitis, this results in a quick improvement of inflammatory symptoms which was confirmed in the results of the beclomethasone group. Oppositely, ectoine acts physically via a mechanism called “preferential exclusion.” In the presence of ectoine, membranes and lipids are protected indirectly. As ectoine is expelled from the surface of proteins and lipids, those are protected by a water shell, thereby increasing the fluidity of membranes and resulting in the preferential formation of the native conformation of proteins [8, 16, 22–25]. This might stabilize mucous membranes such as lining epithelia of the nose, thereby protecting those from invading allergens and reducing allergen-induced inflammations as shown in different model systems [13, 26, 27] and as reported in congress report [28, 29]. It is understood that many allergens which cause allergic rhinitis symptoms have protease activities which act by impairing epithelial barrier function. This in turn results in increased penetration of allergens into nasal mucosa [30]. The barrier stabilizing properties of ectoine may counteract this scenario by improving the epithelial barrier and stabilizing membranes. In allergic rhinitis, this might protect the nasal mucosae from invading allergens, resulting in improvement of symptoms.

The study was intentionally performed as noninterventional study, reflecting the most realistic standard clinical practice and German law. However, this study design forbids randomization of patients, use of placebo, and blinding of study medication. Thus, patients were included independently of their prior medication and no wash-out period had to be kept. All patients had to show a certain degree of symptoms, measured by a minimum TNSS, at study start. Although we believe that valuable results can be drawn from noninterventional trials, one drawback of this study design is the fact that one cannot include a placebo group into the study population. On the other hand, it has been demonstrated that double-blind randomized placebo-controlled trials clearly have their limitations and disadvantages; for example, a comparison of open and controlled study designs in neuroleptic studies indicated that results of well performed open studies can earn more attention. The study design, however, reduces the grade of evidence delivered by the study data from Ib to IIa. On the other hand, it presents a realistic view of the most common clinical practice. Importantly, patient parameters in the current trial seemed to be well balanced between the two groups, with no major differences existing in terms of baseline values at the beginning, demography, history, and symptoms/health status before treatment.

As confirmed in the current study, beclomethasone acts rather quickly, and reduction of nasal symptoms was already observed within the first 12 hours of treatment. Surprisingly, the ectoine nasal spray seems to work comparably quick and results in a clear improvement of symptoms within the same time period of 12 h. Although the percentage of symptom decline was slightly larger in the beclomethasone group (decrease by −47.35%) in comparison to the ectoine group (decrease by −37.44%), decreases were both statistically significant (*P* < 0.001). Within the following treatment days, nasal symptoms decreased further, and at the final visit, TNSS values had decreased by −51.20% in the ectoine group and by 71.49% in the beclomethasone group. The degree of improvement following treatment with nasal corticosteroids corresponds to comparable data from the literature, describing decreases of total nasal symptoms of about 40–85% [31–33]. All those studies reported that corticosteroid treatment of allergic rhinitis worked significantly better than placebo treatment, and although the current study does not include a placebo group, it allows bringing the current results into a broader context.

The decreases of TNSS as assessed by the physicians were confirmed by the patients, with stronger decreases documented in the beclomethasone group in comparison with the ectoine group. In total, patients' baseline TNSS values were lower than the physicians' scores, whereas TNSS values at the end of the study were comparable between physicians and patients assessments. This difference can be explained with the fact that physicians' assessment of baseline values took part prior to treatment, whereas the first patients' documentations of TNSS values were performed at the end of the first treatment day and confirmed the quick onset of action of both treatments.

The aim of the current study was to investigate if ectoine nasal spray is as equally effective as treatment with a glucocorticoid nasal spray. As evaluated with an equivalence test of TNSS values assessed both by physicians and by patients, noninferiority of ectoine versus beclomethasone could not be confirmed. It is noteworthy that the safety profile of both treatments was assessed as good to very good both by investigators and by patients which was underlined by the very low number of adverse events. This goes in line with reports confirming that intranasal glucocorticosteroids can be applied safely [34], even in children and for chronic rhinitis [35]. Positive treatment effects of the current study were also reflected by the results of the quality of life questionnaire which demonstrated improvements in all questioned areas covering daily life activities, general well-being, and the emotional status of patients.

Additional support to the potential efficacy of the ectoine nasal spray comes from similar studies. In a single center, double-blind, placebo-controlled cross-over study consisting of 5 visits involving patients suffering from allergic rhinoconjunctivitis, it could be demonstrated that ectoin nasal spray and eye drops relieved all of the hallmark symptoms of allergic rhinoconjunctivitis with minimal side-effects thereby showing a statistically significant effect over the placebo group. Corresponding data has been presented on a scientific congress [19]. Furthermore, additional noninterventional studies and another placebo-controlled clinical trial involving ectoine containing products in the treatment of allergic rhinitis were analyzed together. Both nasal and ocular symptoms decreased significantly upon treatment with ectoine products. The strength of effects of ectoine products was assessed by comparison of symptom scores on day 7 and baseline values on day 1 with reference products (azelastine, beclomethasone, or cromoglycic acid) or placebo treatment and showed comparable (nasal obstruction and rhinorrhea) or better (nasal itching and sneezing) efficacy of the ectoine products in comparison to control substances ([36], congress report, paper accepted).

Taken together this study supports that allergic rhinitis can be successfully treated with beclomethasone and also it was shown that ectoine nasal spray may be a future treatment option. Whereas efficacy of the pharmaceutically active steroid beclomethasone seems to be superior to that of the natural, barrier stabilizing substance ectoine, with its nonpharmaceutical mode of action, the safety profiles of both treatment groups were comparable. Thus, after proving the hints towards efficacy with further studies, ectoine containing products might become interesting alternative treatment strategies for symptom reduction in allergic rhinitis, particularly for patients seeking nonpharmaceutical treatments, as they contain a natural substance and are free of preservatives. Those alternative treatments are highly demanded but not yet generally recommended [37] and, thus, should be evaluated in more detail in further studies.

## Figures and Tables

**Table 1:** TNSS differences from treatment day 1/site visit 1 (d1/V1) to treatment d14/V2, respectively. Values are given as absolute value differences (abs) or as percentage differences (%).

TNSS difference variable	Equivalence range	TNSS difference of mean values			*P* value
		Value	SD	95% CI	
d14 (abs)	−2.32 ± 0.35	−1.10	0.92	[−2.64; 0.44]	*P* = 0.939
d14 [%]	−39.69 ± 5.95	−26.84	20.86	[−61.96; 8.29]	*P* = 0.938
V2 (abs)	−6.52 ± 0.98	−1.80	0.87	[−3.26; −0.34]	*P* = 0.999
V2 [%]	−71.49 ± 10.72	−20.29	8.91	[−35.25; −5.32]	*P* = 0.999

SD: standard deviation, CI: confidence interval.

**Table 2:** Development of single nasal symptoms during the study. Improvement, deterioration, or unchanged status of single symptoms was assessed by patients and investigators.

	Patients' assessment		Total	Physicians' assessment		Total
	Group 1	Group 2		Group 1	Group 2	
Rhinorrhoea						
Reduced	11 (47.8%)	12 (48.0%)	23 (47.9%)	17 (68.0%)	22 (88.0%)	39 (78.0%)
Unchanged	9 (39.1%)	11 (44.0%)	20 (41.7%)	6 (24.0%)	3 (12.0%)	9 (18.0%)
Deteriorated	3 (13.0%)	2 (8.0%)	5 (10.4%)	2 (8.0%)	0 (0.0%)	2 (4.0%)
Total	**23 (100.0%)**	**25 (100.0%)**	**48 (100.0%)**	**25 (100.0%)**	**25 (100.0%)**	**50 (100.0%)**
Fisher's exact test	*P* = 0.919			*P* = 0.221		

Nasal itching						
Reduced	7 (30.4%)	12 (48.0%)	19 (39.6%)	20 (80.0%)	22 (88.0%)	42 (84.0%)
Unchanged	13 (56.5%)	10 (40.0%)	23 (47.9%)	4 (16.0%)	2 (8.0%)	6 (12.0%)
Deteriorated	3 (13.0%)	3 (13.0%)	6 (12.5%)	1 (4.0%)	1 (4.0%)	2 (4.0%)
Total	**23 (100.0%)**	**25 (100.0%)**	**48 (100.0%)**	**25 (100.0%)**	**25 (100.0%)**	**50 (100.0%)**
Fisher's exact test	*P* = 0.440			*P* = 0.830		

Nasal obstruction						
Reduced	11 (47.8%)	10 (40.0%)	21 (43.8%)	18 (72.0%)	22 (88.0%)	40 (80.0%)
Unchanged	7 (30.4%)	13 (52.0%)	23 (41.7%)	6 (24.0%)	2 (8.0%)	8 (16.0%)
Deteriorated	5 (21.7%)	2 (8.0%)	7 (14.6%)	1 (4.0%)	1 (4.0%)	2 (4.0%)
Total	**23 (100.0%)**	**25 (100.0%)**	**48 (100.0%)**	**25 (100.0%)**	**25 (100.0%)**	**50 (100.0%)**
Fisher's exact test	*P* = 0.258			*P* = 0.347		

Sneezing						
Reduced	5 (21.7%)	12 (48.0%)	17 (35.4%)	18 (72.0%)	23 (92.0%)	41 (82.0%)
Unchanged	10 (43.5%)	11 (44.0%)	21 (43.8%)	3 (12.0%)	2 (8.0%)	5 (10.0%)
Deteriorated	8 (34.8%)	2 (8.0%)	10 (20.8%)	4 (16.0%)	0 (0.0%)	4 (8.0%)
Total	**23 (100.0%)**	**25 (100.0%)**	**48 (100.0%)**	**25 (100.0%)**	**25 (100.0%)**	**50 (100.0%)**
Fisher's exact test	*P* = 0.039			*P* = 0.115		

**Table 3:** Development of ear/palate itching during the study (d1 to d14 or V1 to V2) as assessed by patients and investigators. The total number of patients (% given in brackets) where symptoms were reduced, unchanged, or deteriorated is shown.

Ear/palate itching	Patients' assessment		Total	Physicians' assessment		Total
	Group 1	Group 2		Group 1	Group 2	
Reduced	4 (17.4%)	12 (48.0%)	16 (33.3%)	12 (48.0%)	14 (56.0%)	26 (52.0%)
Unchanged	14 (60.9%)	9 (36.0%)	23 (47.9%)	12 (48.0%)	8 (32.0%)	20 (40.0%)
Deteriorated	5 (21.7%)	4 (16.0%)	9 (18.8%)	1 (4.0%)	3 (12.0%)	4 (8.0%)
Total	**23 (100.0%)**	**25 (100.0%)**	**48 (100.0%)**	**25 (100.0%)**	**25 (100.0%)**	**50 (100.0%)**
Fisher's exact test	*P* = 0.088			*P* = 0.357		

**Table 4:** Results (changes from V1 to V2) of the quality of life questionnaire documented by physicians.

		Group 1	Group 2	Total
Frequency of tissue use				
*P* = 0.568	Reduced	12 (48.0%)	15 (60.0%)	27 (54.0%)
	Unchanged	11 (44.0%)	7 (28.0%)	18 (36.0%)
	Increased	2 (8.0%)	3 (12.0%)	5 (10.0%)
	Total	**25 (100.0%)**	**25 (100.0%)**	**50 (100.0%)**

Rubbing eyes and nose				
*P* = 0.999	Reduced	14 (56.0%)	14 (56.0%)	28 (56.0%)
	Unchanged	8 (32.0%)	7 (28.0%)	15 (30.0%)
	Increased	3 (12.0%)	4 (16.0%)	7 (14.0%)
	Total	**25 (100.0%)**	**25 (100.0%)**	**50 (100.0%)**

Frequency of brushing of nose				
*P* = 0.008	Reduced	12 (48.0%)	21 (84.0%)	33 (66.0%)
	Unchanged	8 (32.0%)	4 (16.0%)	12 (24.0%)
	Increased	5 (20.0%)	0 (0.0%)	5 (10.0%)
	Total	**25 (100.0%)**	**25 (100.0%)**	**50 (100.0%)**

Bad sleep				
*P* = 0.878	Reduced	15 (60.0%)	17 (68.0%)	32 (64.0%)
	Unchanged	9 (36.0%)	7 (28.0%)	16 (32.0%)
	Increased	1 (4.0%)	1 (4.0%)	2 (4.0%)
Gesamt	Total	**25 (100.0%)**	**25 (100.0%)**	**50 (100.0%)**

Bad work performance				
*P* = 0.328	Reduced	15 (60.0%)	20 (80.0%)	35 (70.0%)
	Unchanged	7 (28.0%)	4 (16.0%)	11 (22.0%)
	Increased	3 (12.0%)	1 (4.0%)	4 (8.0%)
	Total	**25 (100.0%)**	**25 (100.0%)**	**50 (100.0%)**

Fatigue				
*P* = 0.690	Reduced	16 (64.0%)	15 (60.0%)	31 (62.0%)
	Unchanged	8 (32.0%)	7 (28.0%)	15 (30.0%)
	Increased	1 (4.0%)	3 (12.0%)	4 (8.0%)
	Total	**25 (100.0%)**	**25 (100.0%)**	**50 (100.0%)**

Thirst				
*P* = 0.178	Reduced	10 (40.0%)	14 (56.0%)	24 (48.0%)
	Unchanged	8 (32.0%)	9 (36.0%)	17 (34.0%)
	Increased	7 (28.0%)	2 (8.0%)	9 (18.0%)
	Total	**25 (100.0%)**	**25 (100.0%)**	**50 (100.0%)**

Lack of concentration				
*P* = 0.389	Reduced	10 (40.0%)	15 (60.0%)	25 (50.0%)
	Unchanged	13 (52.0%)	8 (32.0%)	21 (42.0%)
	Increased	2 (8.0%)	2 (8.0%)	4 (8.0%)
	Total	**25 (100.0%)**	**25 (100.0%)**	**50 (100.0%)**

General well-being				
*P* = 0.462	Reduced	20 (80.0%)	16 (64.0%)	36 (72.0%)
	Unchanged	4 (16.0%)	6 (24.0%)	10 (20.0%)
	Increased	1 (4.0%)	3 (12.0%)	4 (8.0%)
	Total	**25 (100.0%)**	**25 (100.0%)**	**50 (100.0%)**

Headache				
*P* = 0.081	Reduced	16 (64.0%)	10 (40.0%)	26 (52.0%)
	Unchanged	8 (32.0%)	9 (36.0%)	17 (34.0%)
	Increased	1 (4.0%)	6 (24.0%)	7 (14.0%)
	Total	**25 (100.0%)**	**25 (100.0%)**	**50 (100.0%)**

Bad temper				
*P* = 0.549	Reduced	13 (52.0%)	17 (68.0%)	30 (60.0%)
	Unchanged	8 (32.0%)	6 (24.0%)	14 (28.0%)
	Increased	4 (16.0%)	2 (8.0%)	6 (12.0%)
	Total	**25 (100.0%)**	**25 (100.0%)**	**50 (100.0%)**

General disconcertment				
*P* = 0.099	Reduced	12 (48.0%)	19 (76.0%)	31 (62.0%)
	Unchanged	11 (44.0%)	6 (24.0%)	17 (34.0%)
	Increased	2 (8.0%)	0 (0.0%)	2 (4.0%)
	Total	**25 (100.0%)**	**25 (100.0%)**	**50 (100.0%)**

Frustration				
*P* = 0.195	Reduced	10 (40.0%)	16 (64.0%)	26 (52.0%)
	Unchanged	14 (56.0%)	8 (32.0%)	22 (44.0%)
	Increased	1 (4.0%)	1 (4.0%)	2 (4.0%)
	Total	**25 (100.0%)**	**25 (100.0%)**	**50 (100.0%)**

Reactions of others to the allergy				
*P* = 0.377	Reduced	7 (28.0%)	10 (40.0%)	17 (34.0%)
	Unchanged	18 (72.0%)	14 (56.0%)	32 (64.0%)
	Increased	0 (0.0%)	1 (4.0%)	1 (2.0%)
	Total	**25 (100.0%)**	**25 (100.0%)**	**50 (100.0%)**

In addition to the analysis described above, total decreases of scores of the quality of life questionnaires were analyzed. As depicted in Figure 5 and Figure 6, treatment resulted in decreases of all questioned parameters, thus indicating that all bothersome points which were covered in the questionnaire of life had improved during treatment.

**Table 5:** Adverse events during the study.

Description AE	Treatment group	Relationship
Headache	Ectoine	Highly unlikely
Headache	Ectoine	Highly unlikely
Burning of nose	Beclomethasone	Probably
